# Supplementary material for: A multi-omics integrative approach unravels novel genes and pathways associated with senescence escape after targeted therapy in NRAS mutant melanoma
Source: Cancer Gene Ther. 2023 Jul 7;30(10):1330–45. doi: 10.1038/s41417-023-00640-z (PMC10581906; doi:10.1038/s41417-023-00640-z)
Supplement: Supplementary file 1 — Supplementary material [file 41417_2023_640_MOESM1_ESM.pdf]

Supplementary Figures

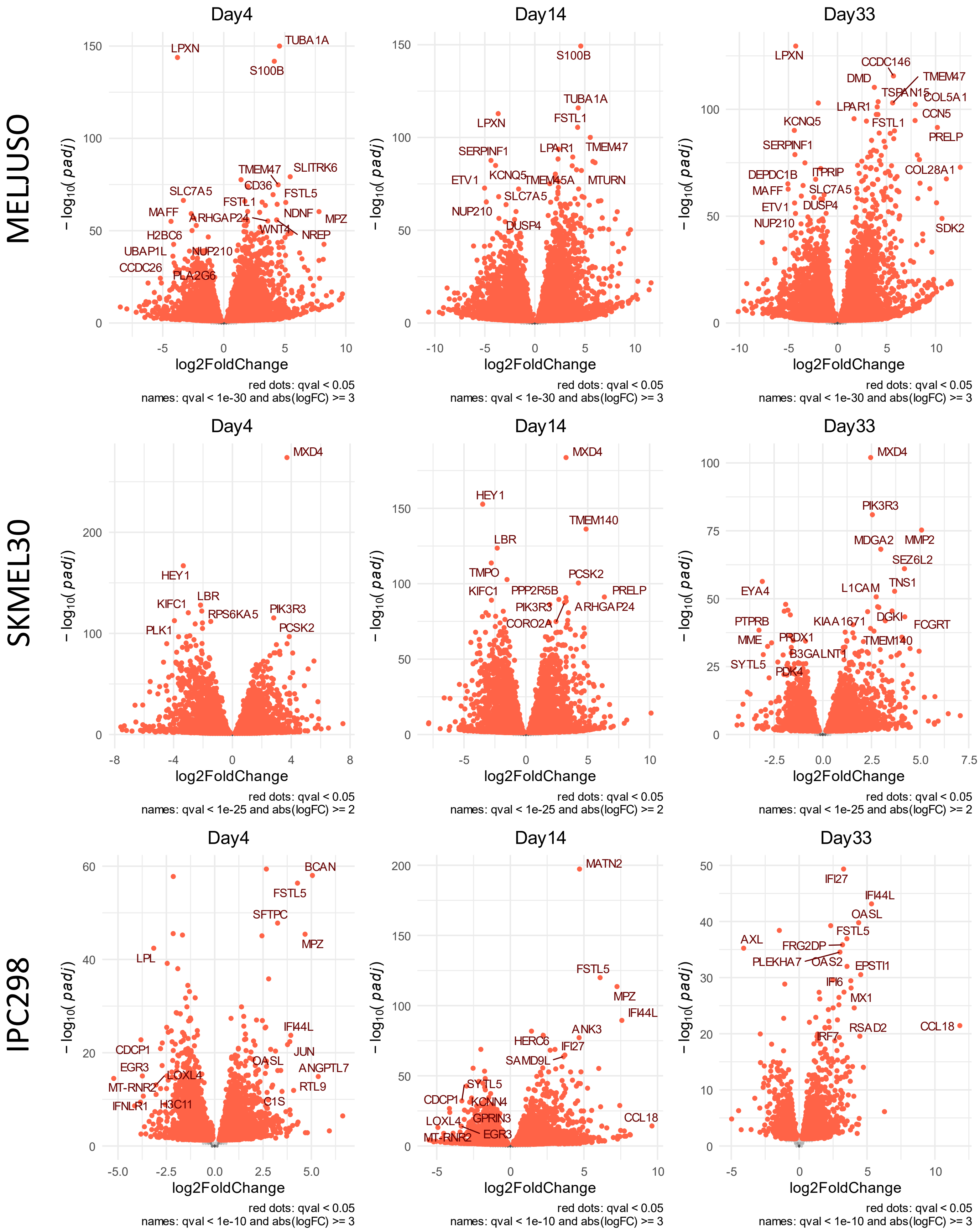

Supplementary Figure 1. Volcano plots for the total RNA-seq. MELJUSO, SKMEL30 and IPC298 cell lines were treated with CDK4/6i and MEKi.

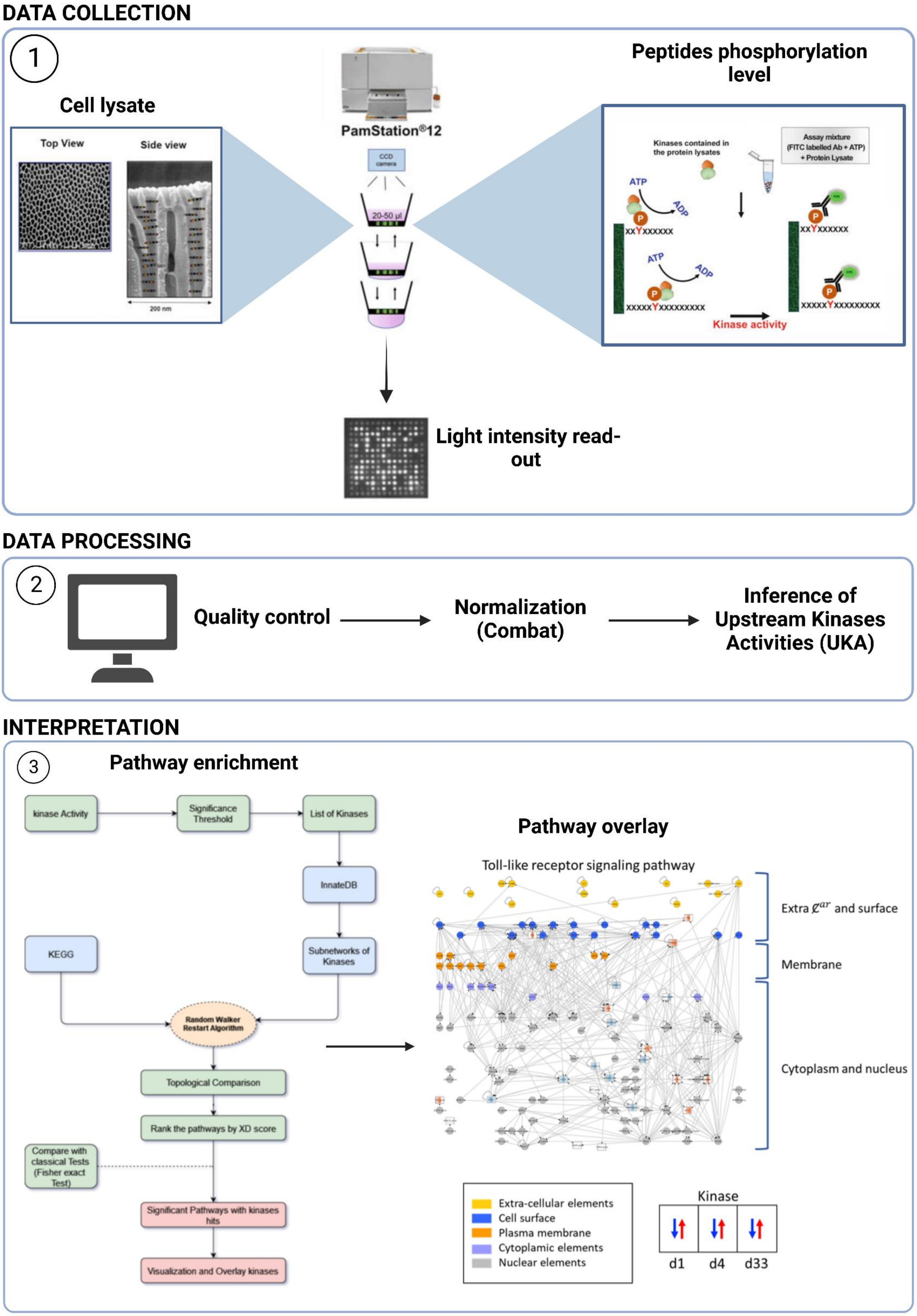

Supplementary Figure 2. Pamgene data acquisition, processing and interpretation workflow.

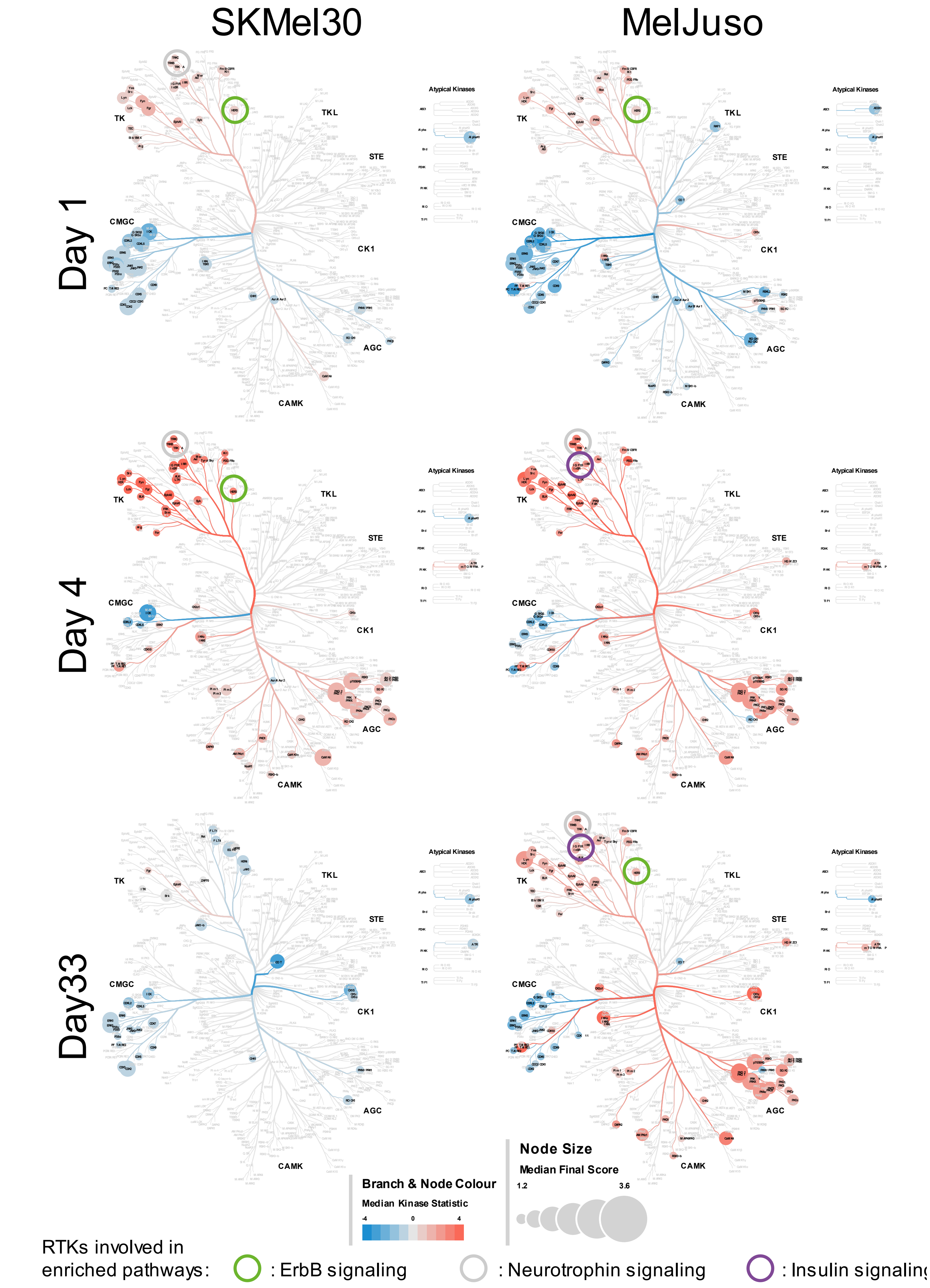

Supplementary Figure 3. Phylogenetic trees representing the kinome responses to MEKi and CDK4/6i. The size of the leafs represents the "Median Final Score", a score greater than 1.2 indicates a significant change between conditions. The color of the branchs and leafs shows the "Median Kinase Statistic" which is the difference in kinase activity. Circles highlight RTKs contributing to enriched pathways. SKMEL30 and MELJUSO cell lines show similar responses at early time points but not at 33 days.

MELJUSO

ErbB signaling pathway

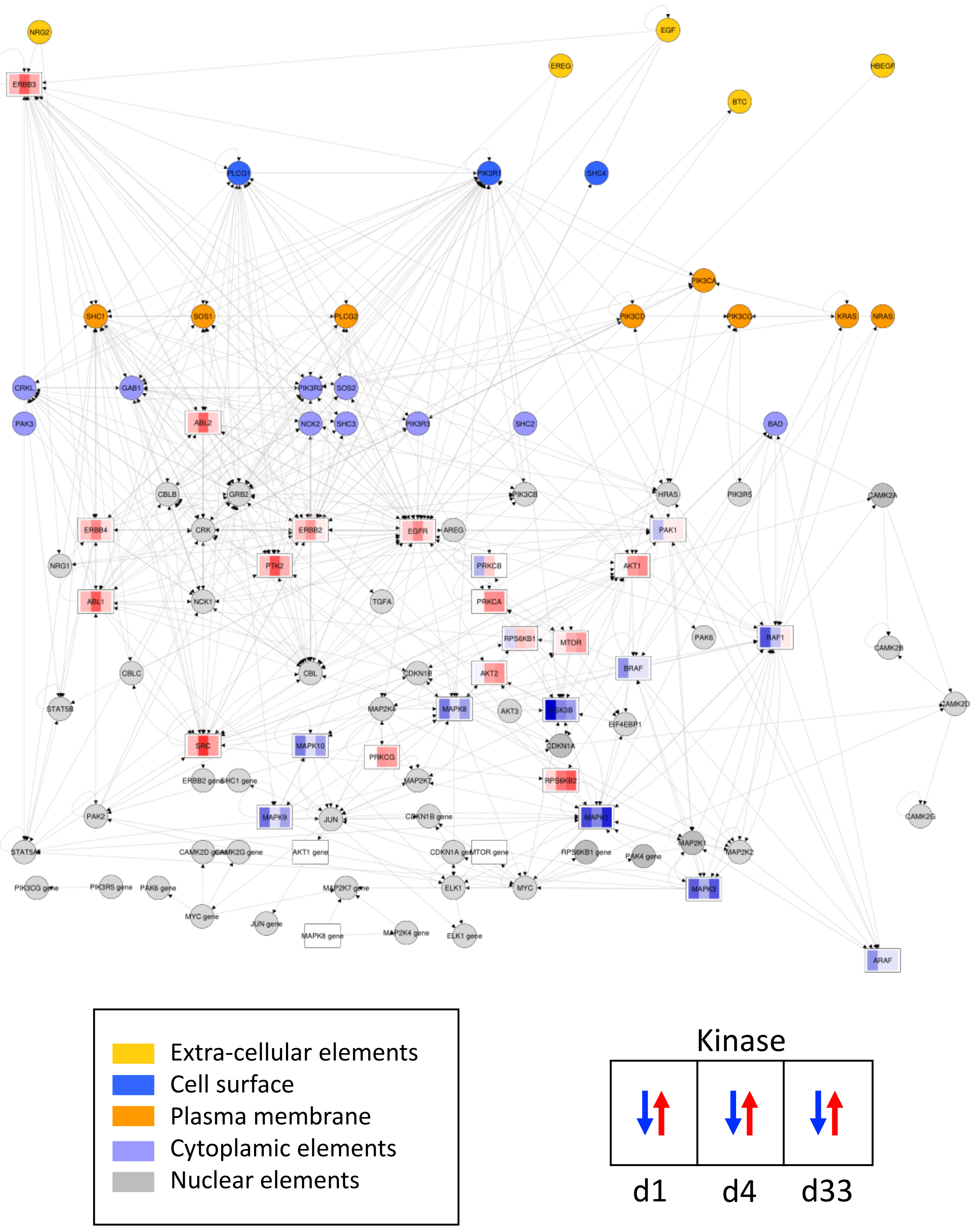

Supplementary Figure 4. Pathway representation of ErbB signaling in MELJUSO cell line. The color of the nodes represents the cellular localization while kinases are illustrated as rectangles with stripes showing their activity at day 1, day4 and day 33.

SKMEL30

ErbB signaling pathway

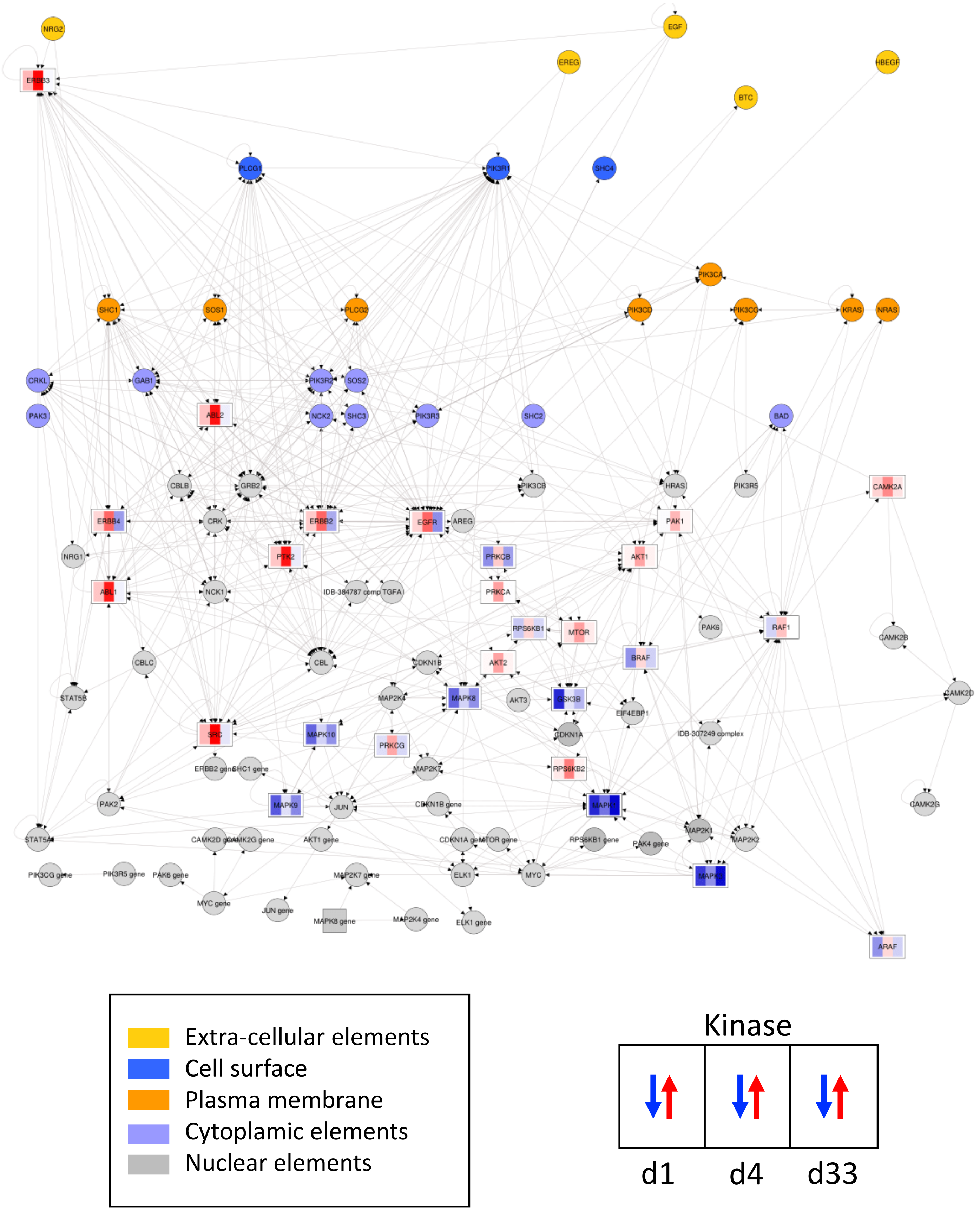

Supplementary Figure 5. Pathway representation of ErbB signaling in SKMEL30 cell line. The color of the nodes represents the cellular localization while kinases are illustrated as rectangles with stripes showing their activity at day 1, day4 and day 33.

MELJUSO

Neurotrophin signaling pathway

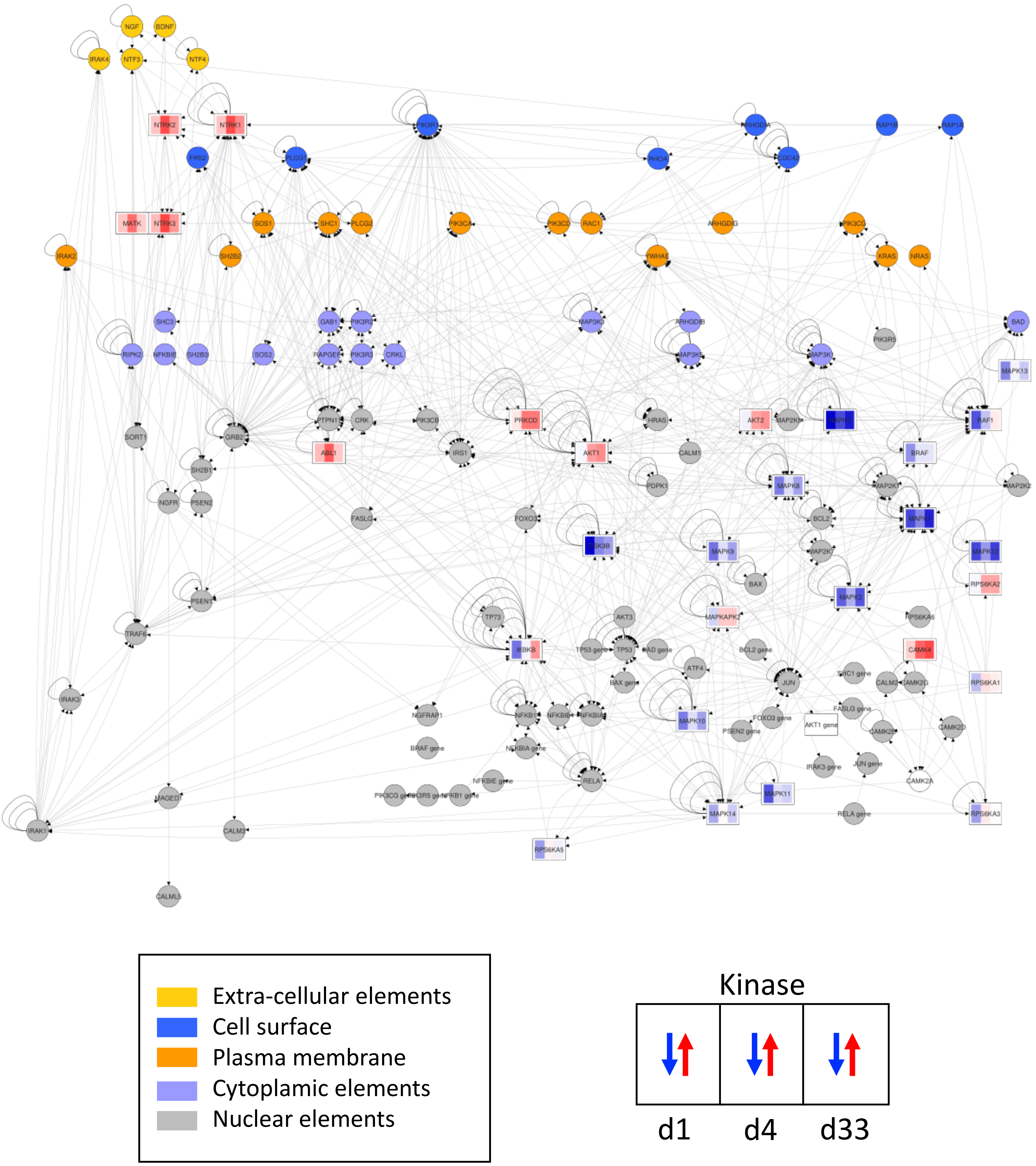

Supplementary Figure 6. Pathway representation of Neurotrophin signaling in MELJUSO cell line. The color of the nodes represents the cellular localization while kinases are illustrated as rectangles with stripes showing their activity at day 1, day4 and day 33.

SKMEL30

Neurotrophin signaling pathway

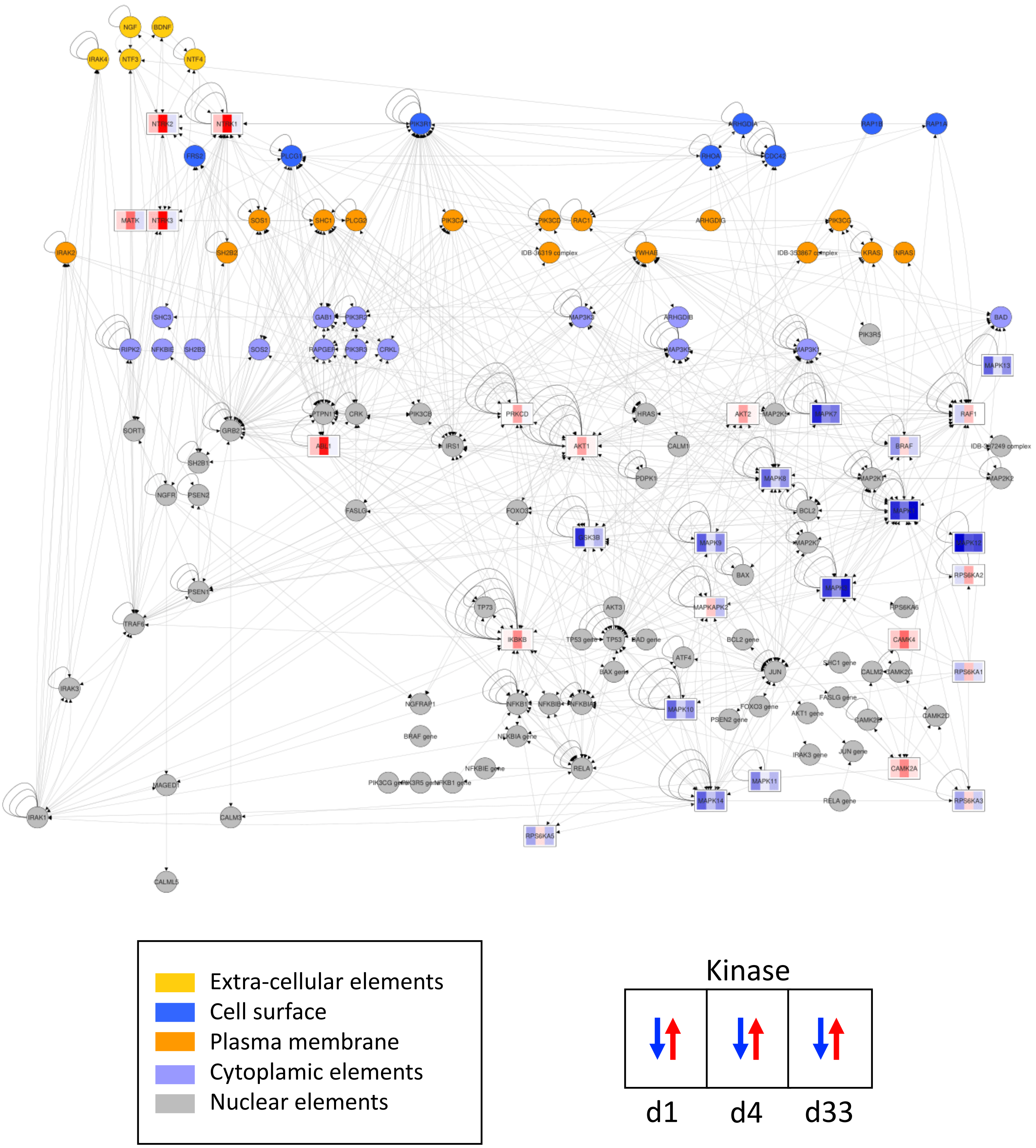

Supplementary Figure 7. Pathway representation of Neurotrophin signaling in SKMEL30 cell line. The color of the nodes represents the cellular localization while kinases are illustrated as rectangles with stripes showing their activity at day 1, day4 and day 33.

MELJUSO

Insulin signaling pathway

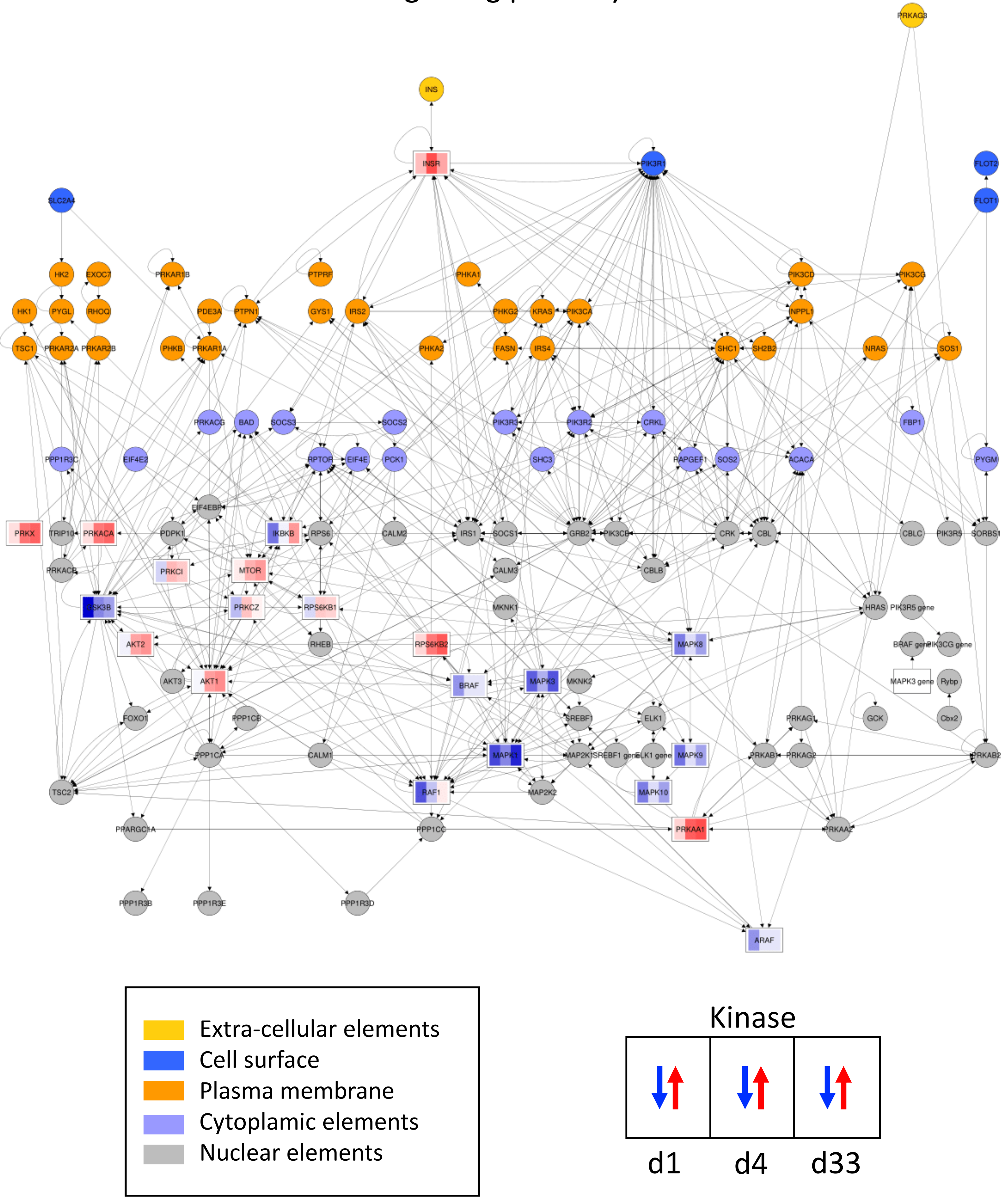

Supplementary Figure 8. Pathway representation of Insulin signaling in MELJUSO cell line. The color of the nodes represents the cellular localization while kinases are illustrated as rectangles with stripes showing their activity at day 1, day4 and day 33.

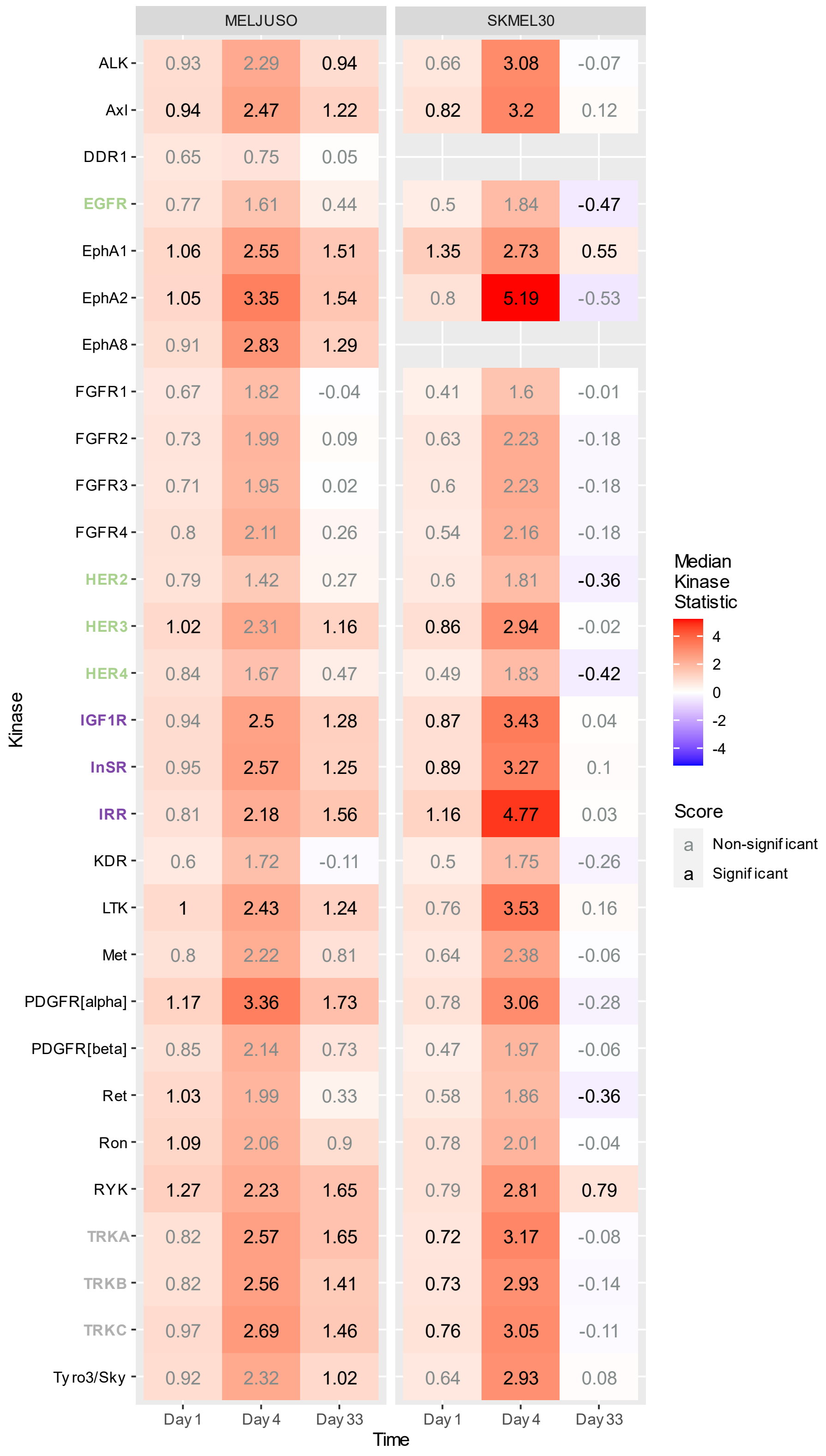

Supplementary Figure 9. Upregulation of RTKs upon MEKi and CDK4/6i. The heatmap represents the "Median Kinase Statistic" for kinases, significant values are represented in black. RTKs are colored according to the enriched pathways in which they are involved

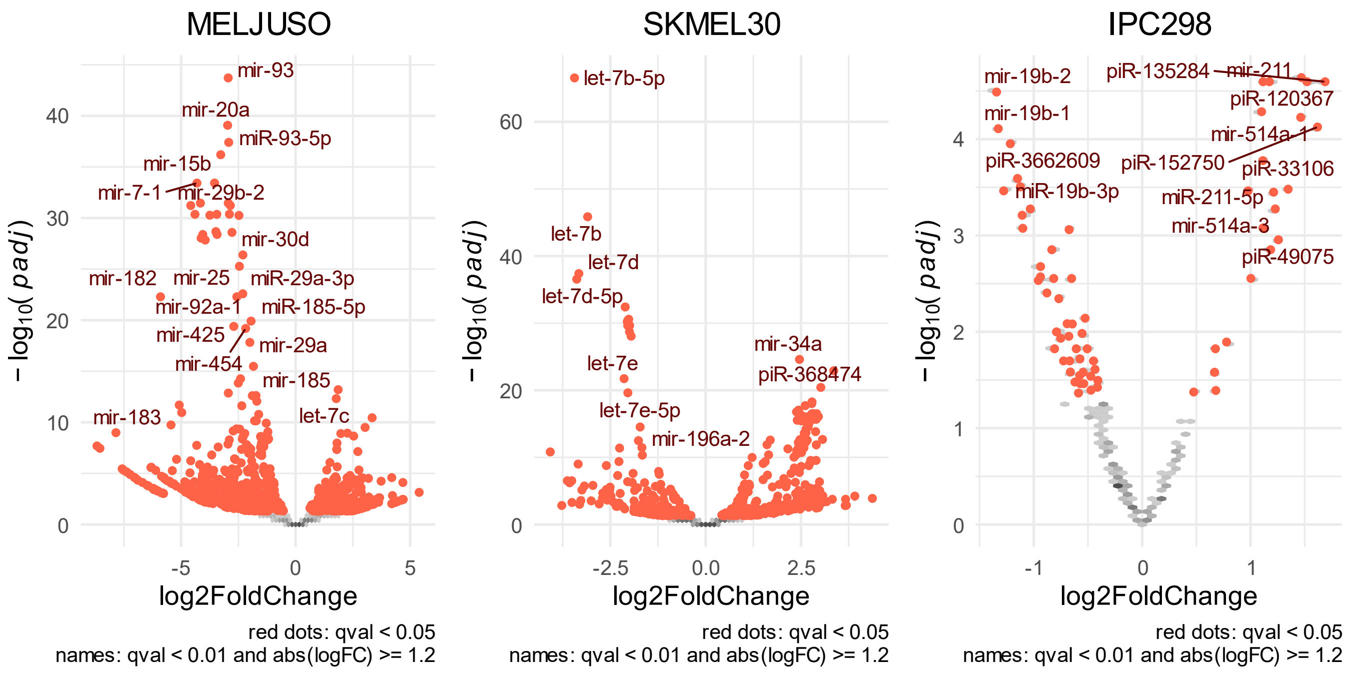

Supplementary Figure 10. Volcano plots for the small RNA-seq. MELJUSO, SKMEL30 and IPC298 cell lines were treated with CDK4/6i and MEKi.

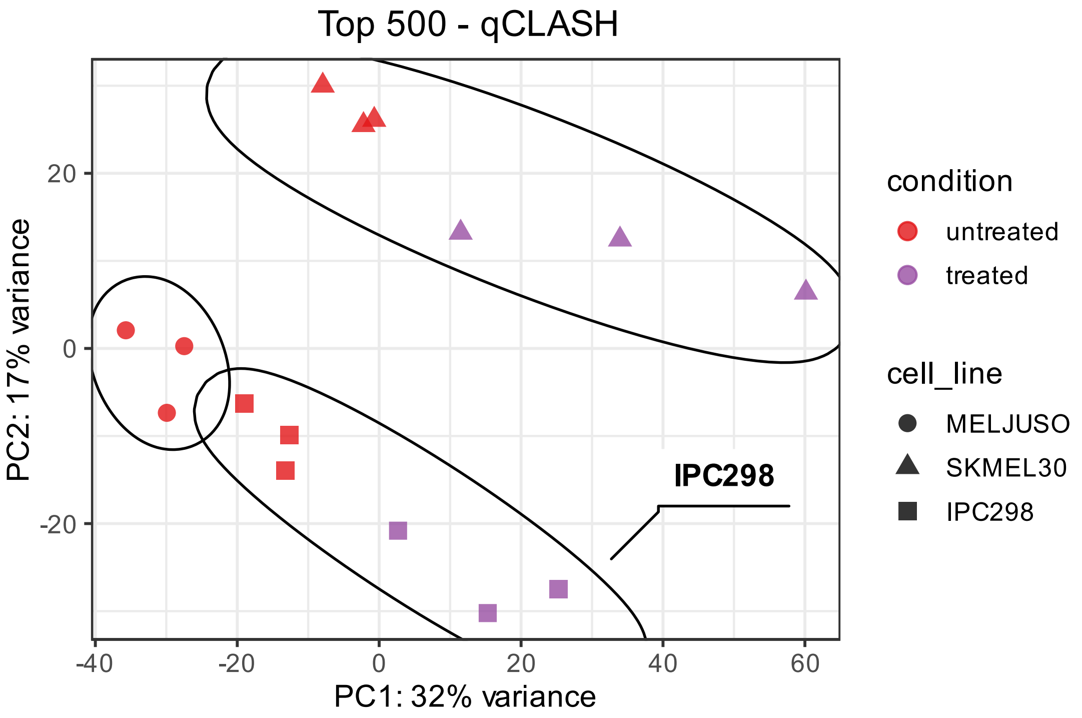

Supplementary Figure 11. Principal Component analysis (PCA) for the top 500 most detected interactions. PCA clearly distinguishes cell lines and conditions.

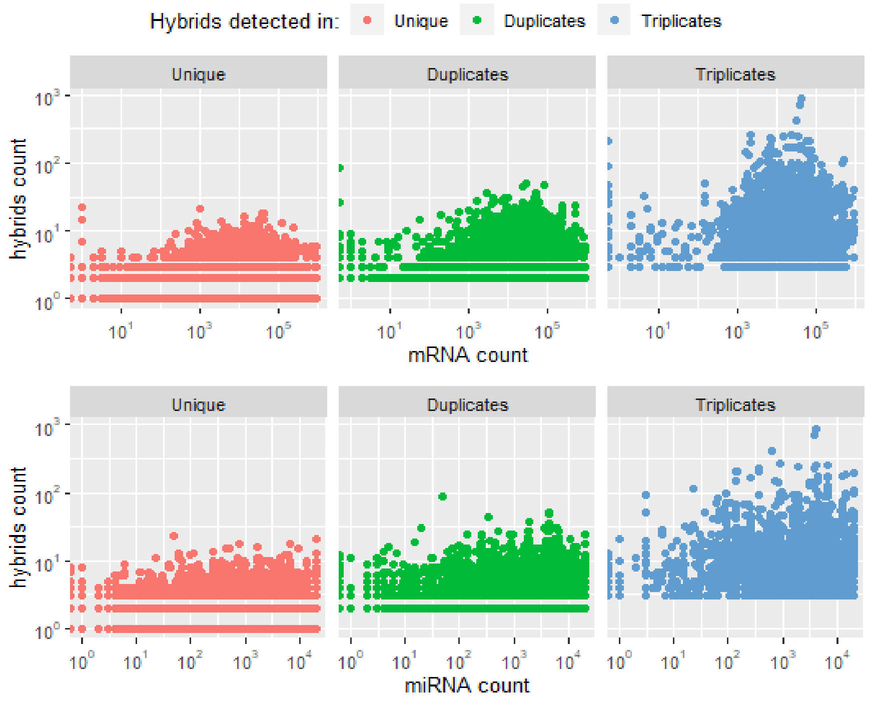

Supplementary Figure 12. miRNA/mRNA vs hybrids count detected in one, two or three biological replicates. Interaction detected in triplicates are associated with greater expression values.

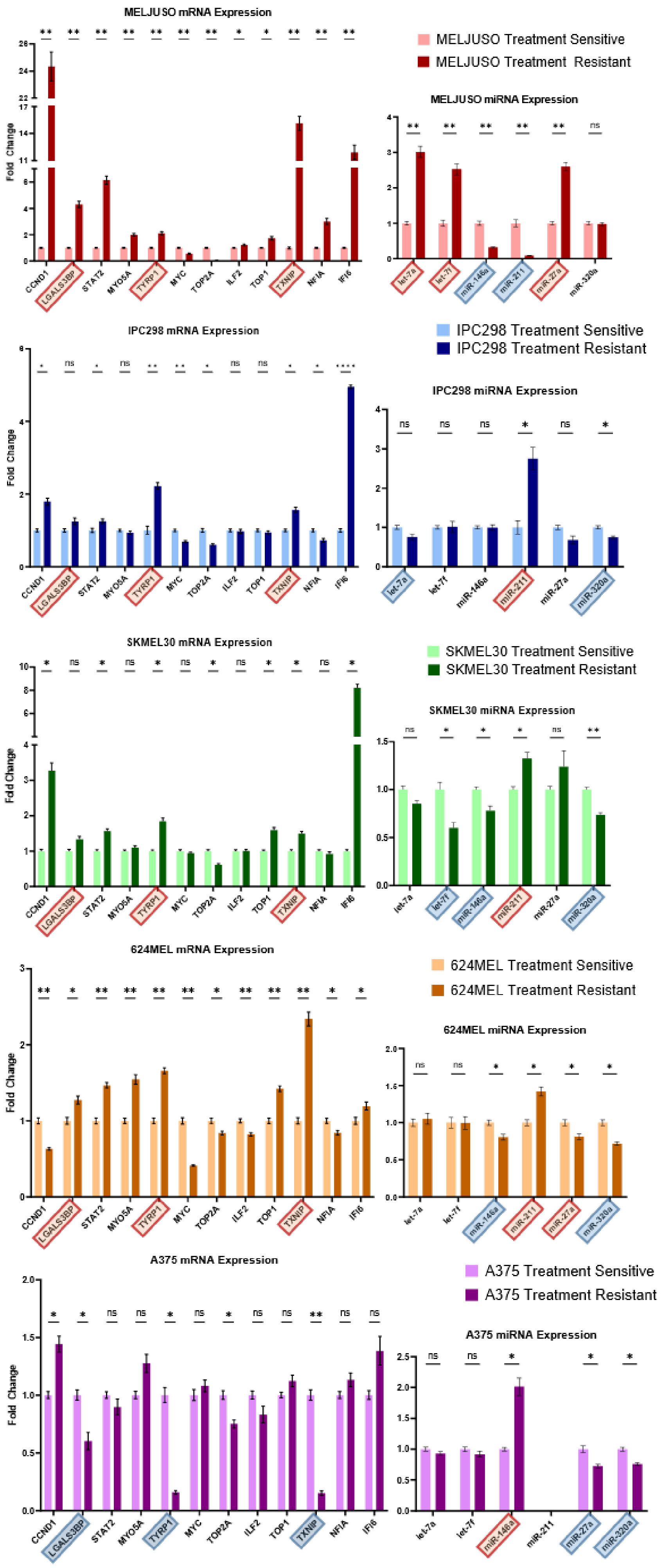

Supplementary Figure 13. miRNA/mRNA expression detected by qPCR across three NRAS and two BRAF cell lines. Statistical significance was calculated considering biological replicates (n=3) using multiple welch's t-test and an FDR of 0.01. Error bars represent Standard Error of the Mean (SEM). Red rectangles highlight upregulated elements and blue rectangles, downregulated elements.

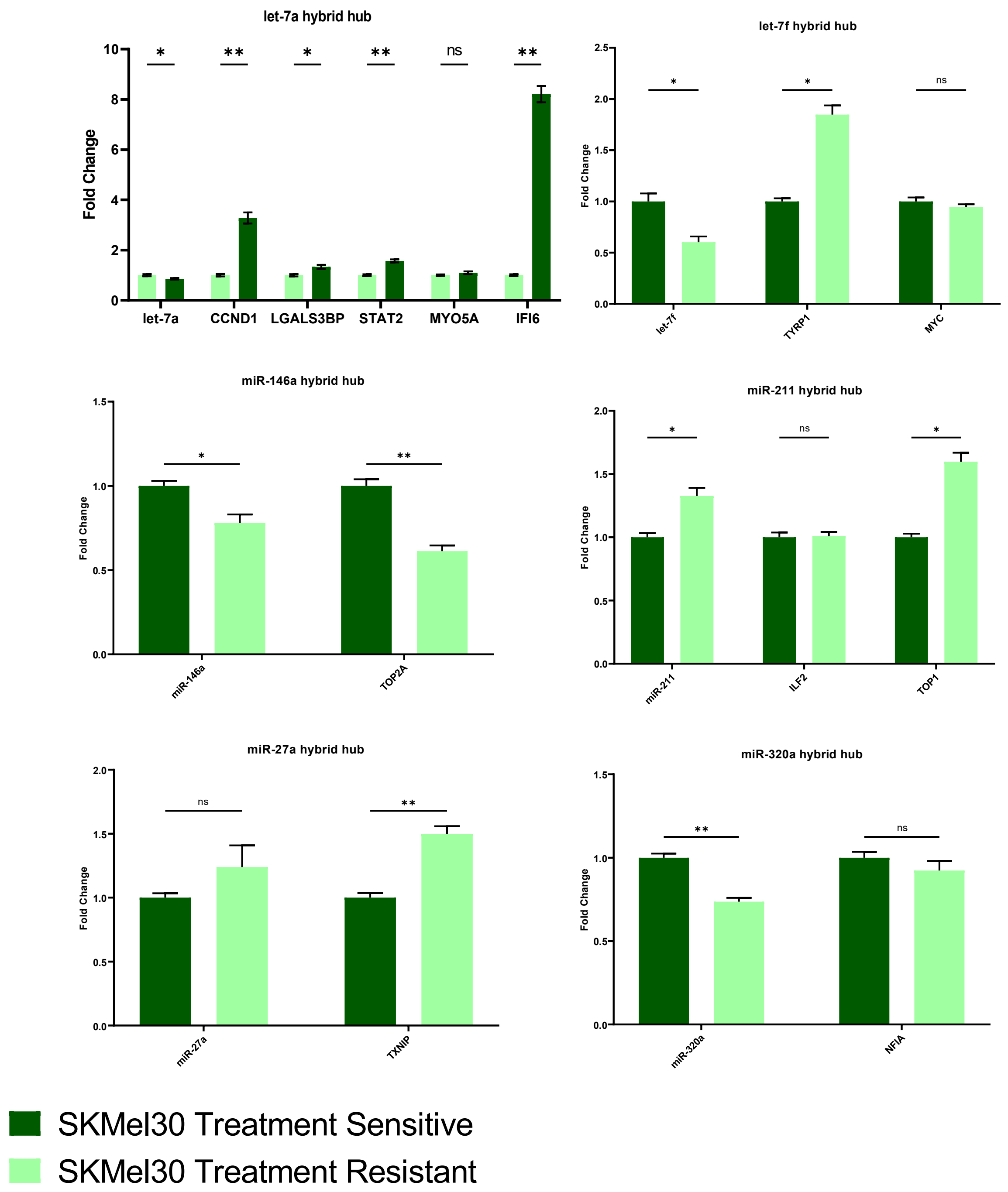

Supplementary Figure 14. Expression of miRNAs and mRNAs detected by qPCR in let-7a, let-7f, miR-27a, miR-146a, miR-211 and miR-320a hubs in SKMEL30.

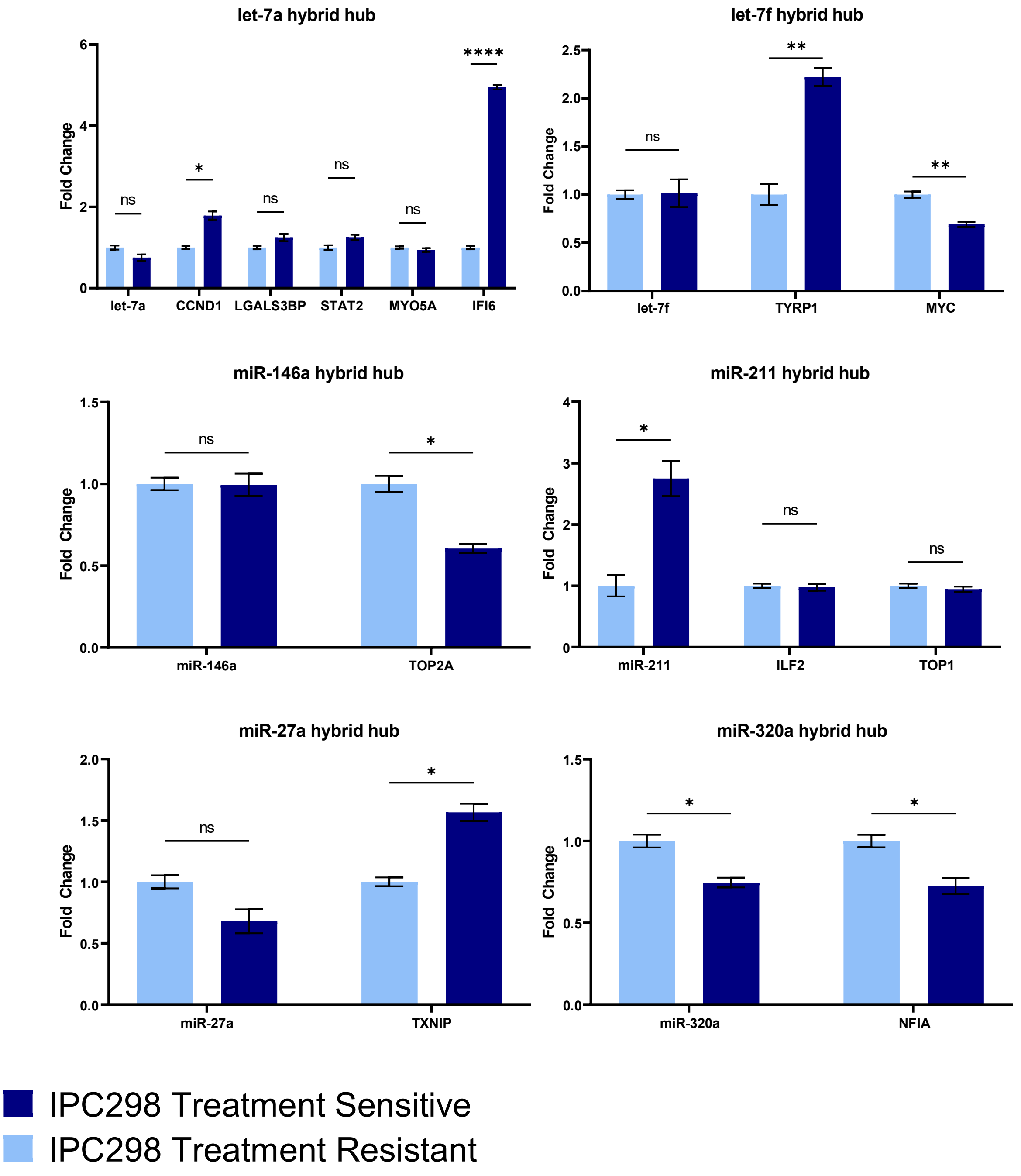

Supplementary Figure 15. Expression of miRNAs and mRNAs detected by qPCR in let-7a, let-7f, miR-27a, miR-146a, miR-211 and miR-320a hubs in IPC298.

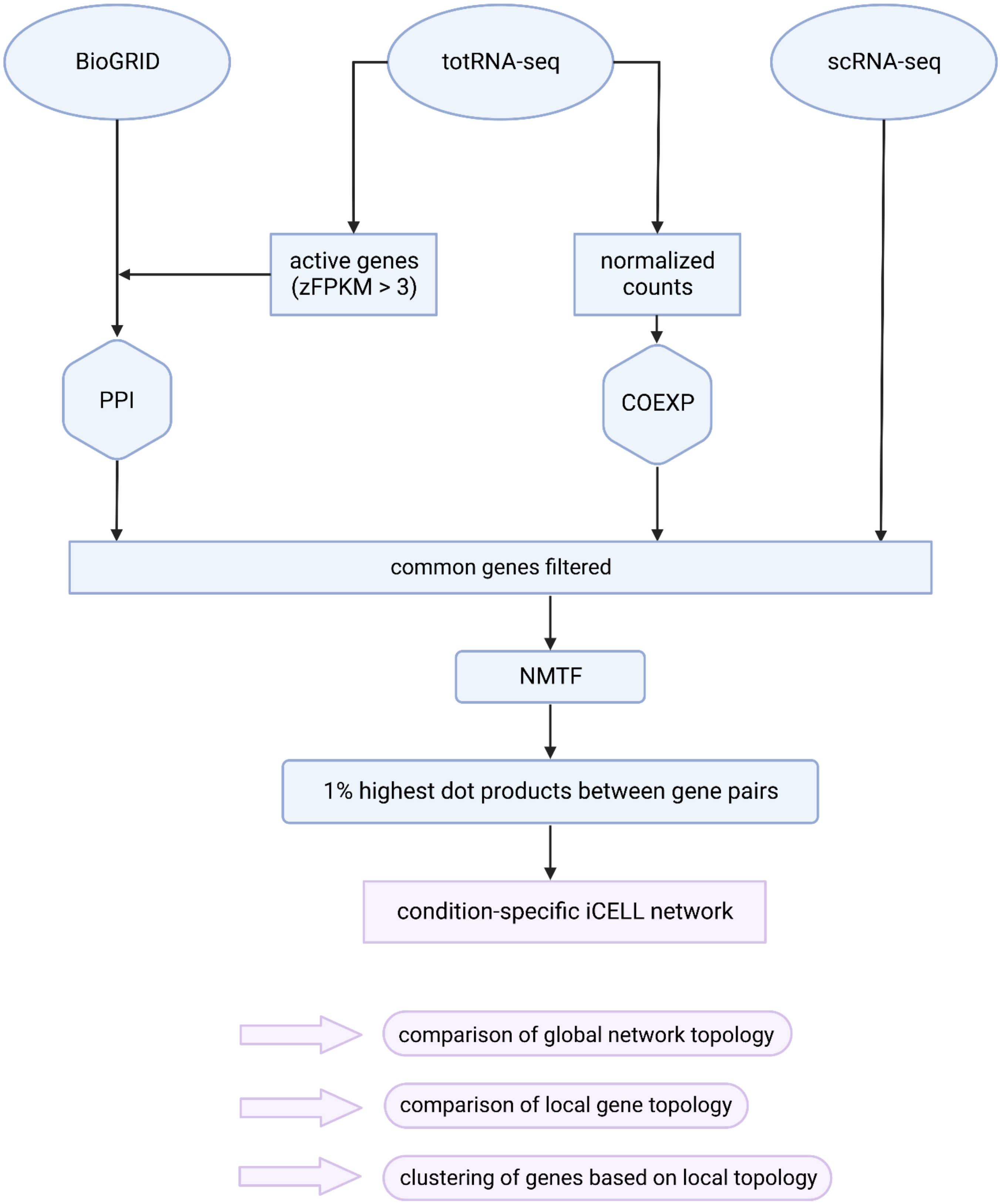

Supplementary Figure 16. Workflow of the data integration, construction an investigation of the iCELL networks.

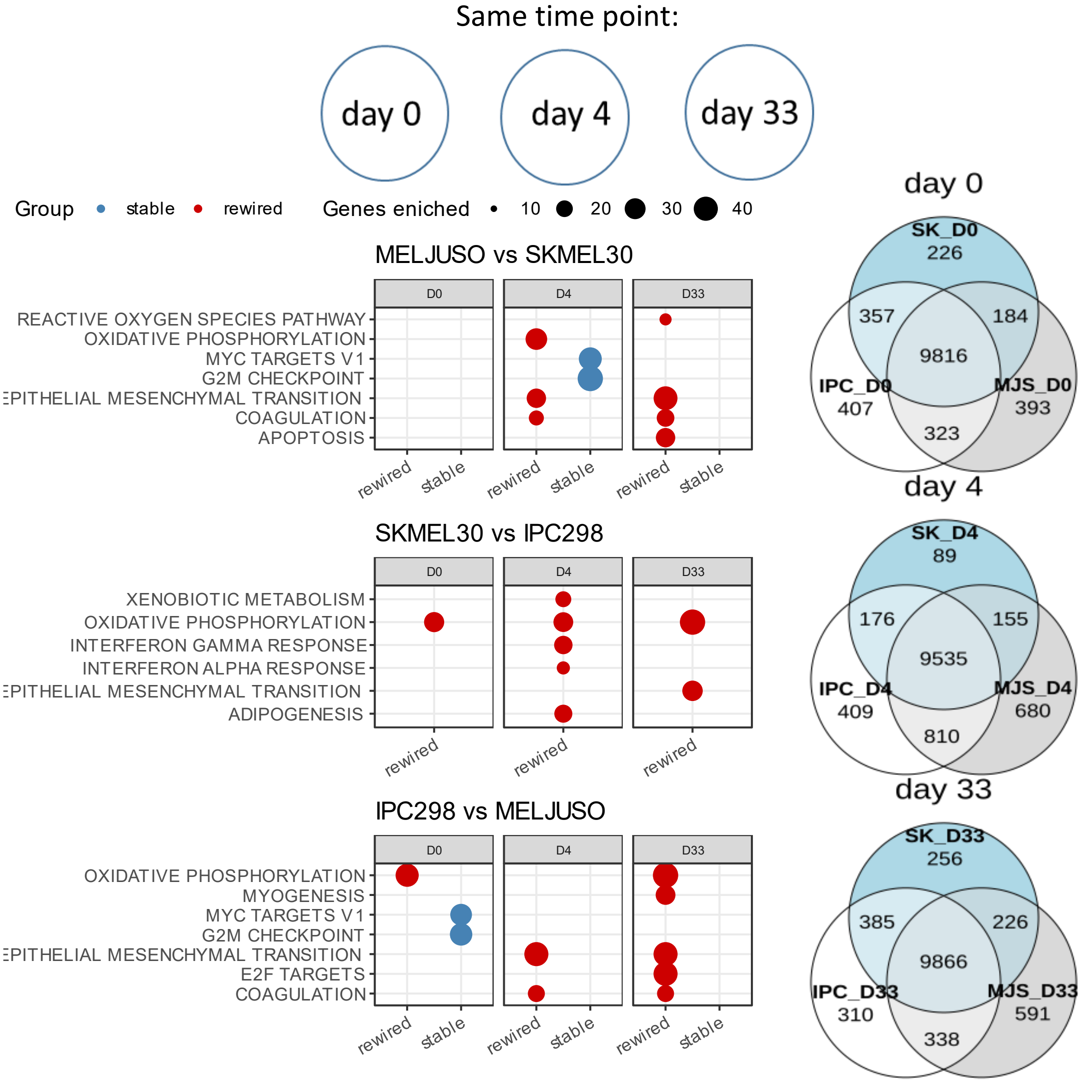

Supplementary Figure 17. Comparison of gene topology using Graphlet Degree Signature Similarity (GDSS) for matching time points. ORA was performed on the top 10% most "stable" or "perturbated" genes. Venn diagrams represent genes overlap between conditions.

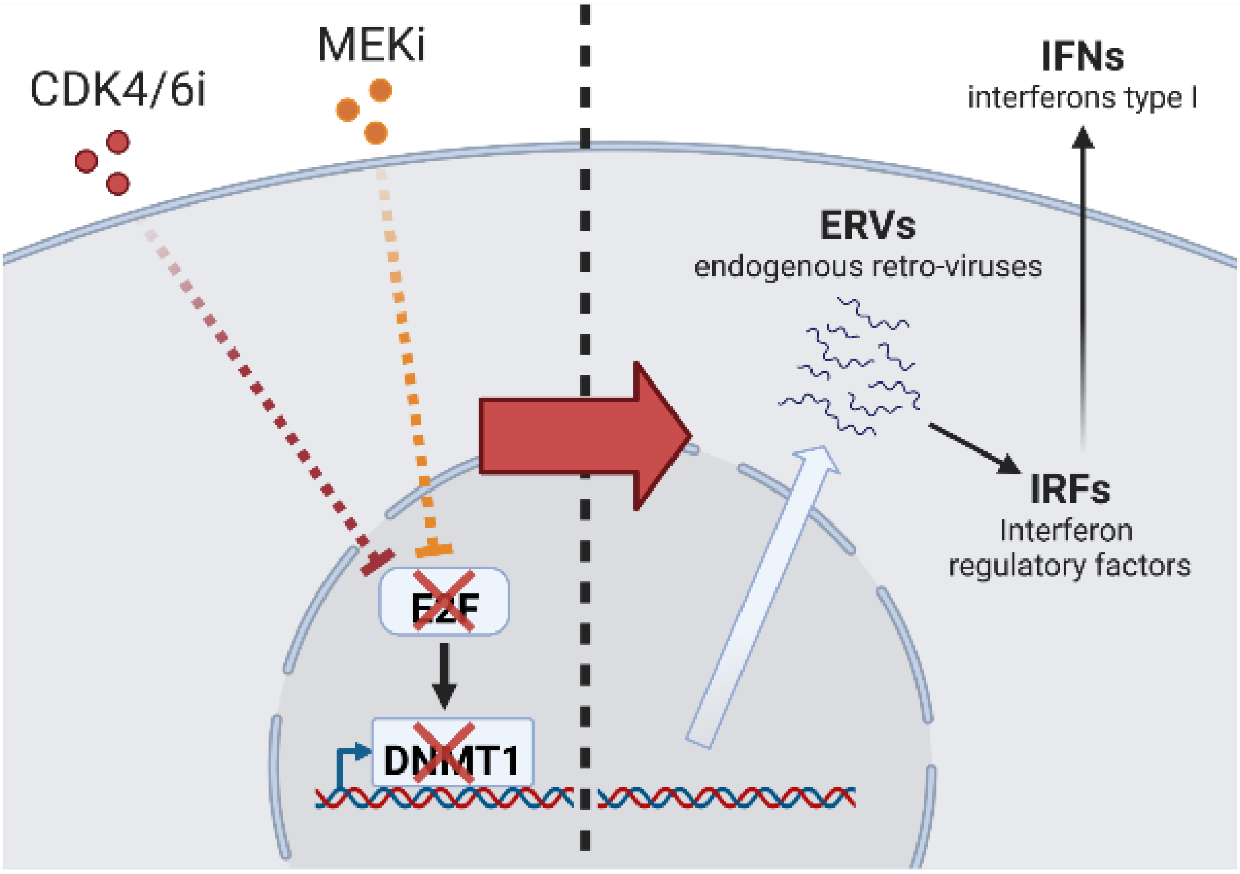

Supplementary Figure 18. Activation of the viral mimicry upon MEKi and CDK4/6i. MEK and CDK4/6 inhibitors converge on the E2F2 transcription factor. E2F2 regulates the DNA methyltransferase 1 enzyme (DNMT1) responsible for maintaining the DNA methylation profil. The downregulation of DNMT1 leads to a global hypomethylation of the genome and allows the expression of Endogenous retroviruses (ERVs). These transcripts hybridise in the cytoplasm and form dsRNA reminiscent of a viral infection. Cytosolic Pattern Recognition Receptors (PRR) such as RIG-I or MDA5 or endosomal one such as TLR3 then activate the innate immune system leading to an interferon signaling.

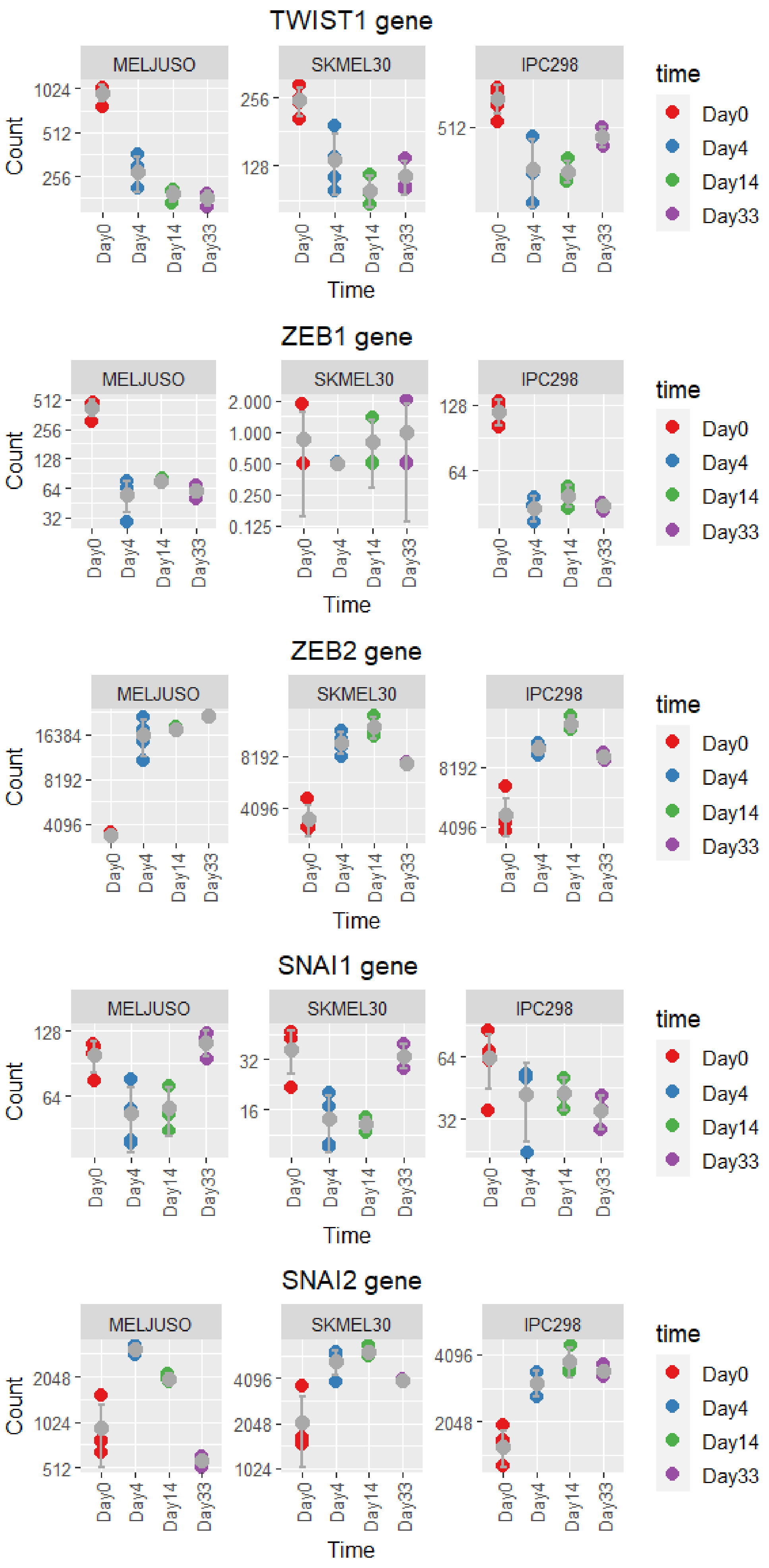

Supplementary Figure 19. Expression of common regulators of senescence and EMT as listed by (Mir Mohd Faheem et al. 2020) upon MEKi and CDK4/6i.

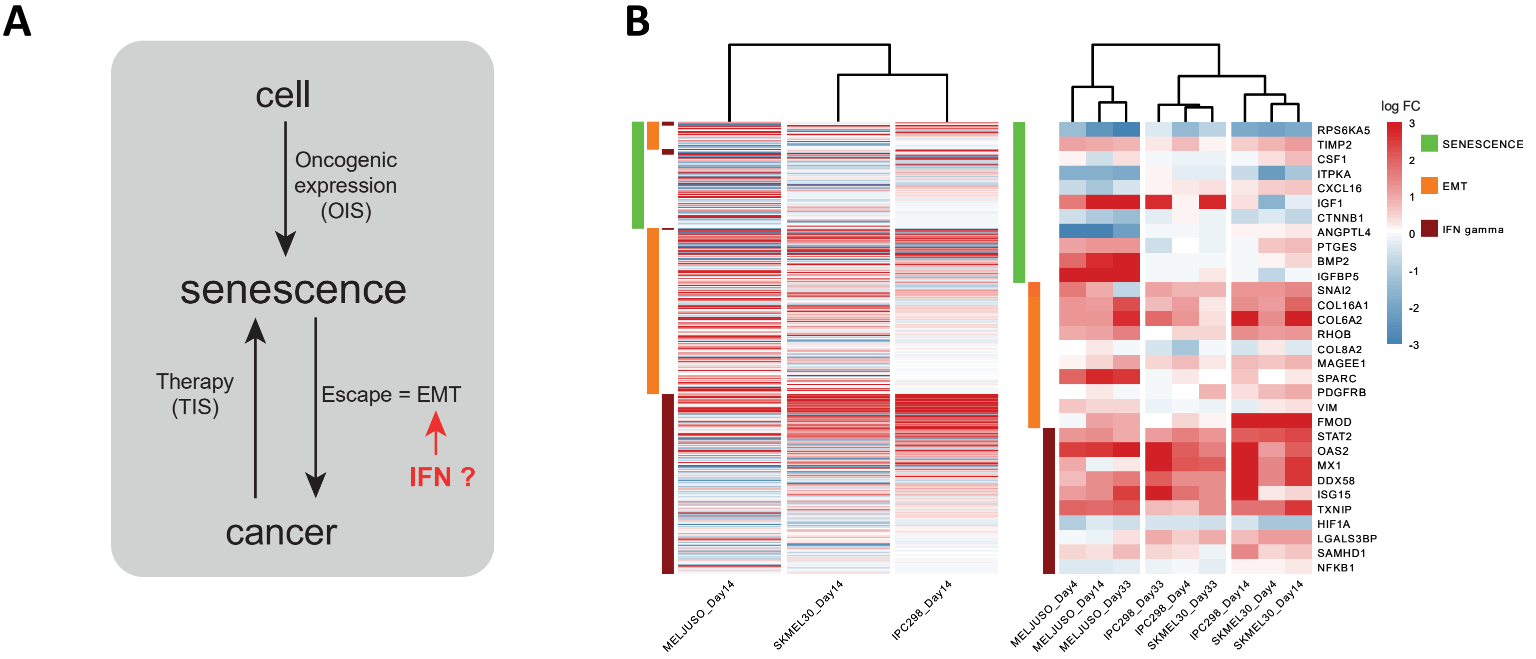

Supplementary Figure 20. Trend in expression between the “EMT” and IFN gamma gene sets from the hallmarks MSigDB and a recent “senescence” gene list. A. Scheme representing the relationships between the different gene sets. B. Heatmap for the "INTERFERON GAMMA RESPONSE" and "EPITHELIAL MESENCHYMAL TRANSITION" hallmarks gene sets and the “SenMayo” gene list [28].

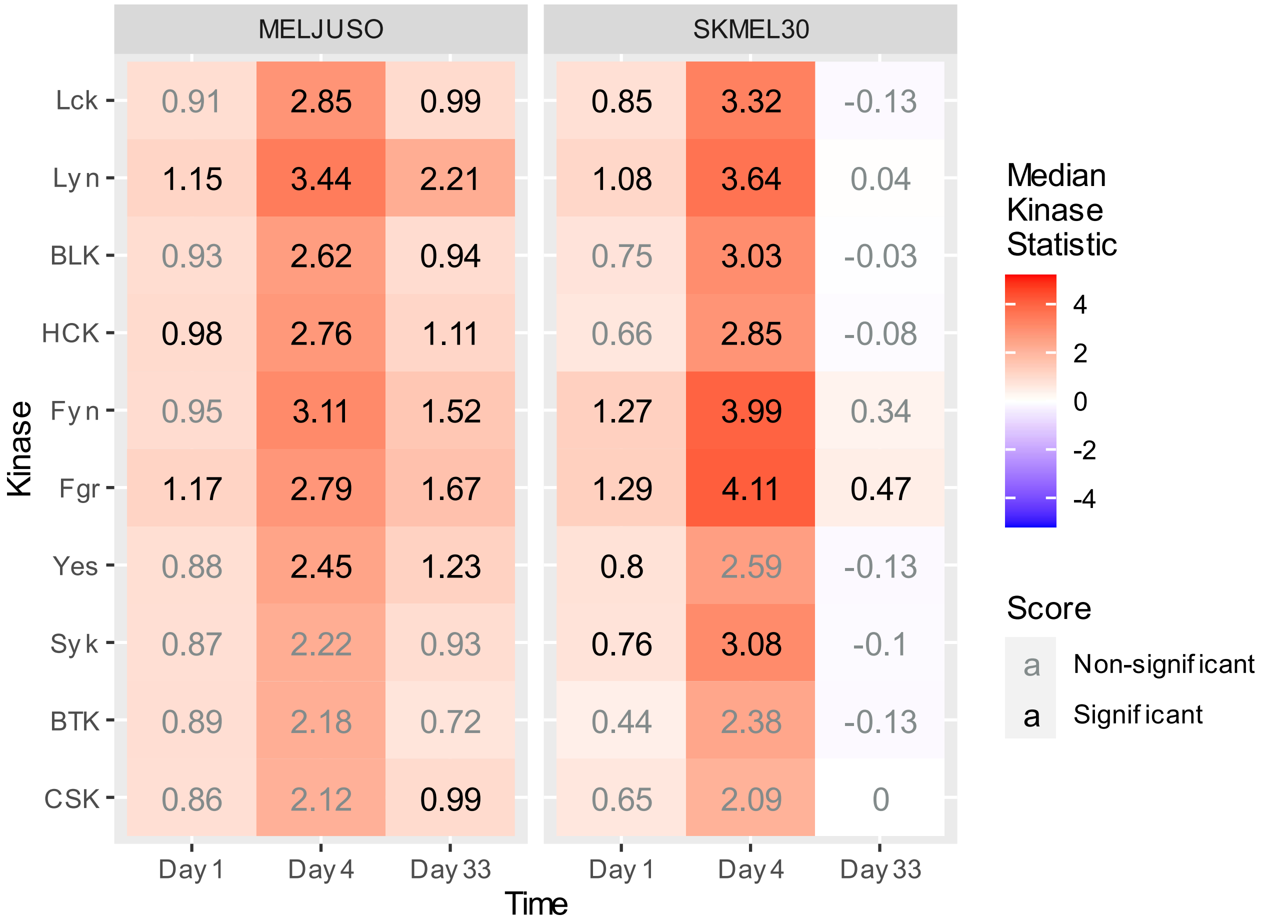

Supplementary Figure 21. Activation of Src Family Kinases (SFKs). The heatmap represents the "Median Kinase Statistic" for kinases, significant values are represented in black.

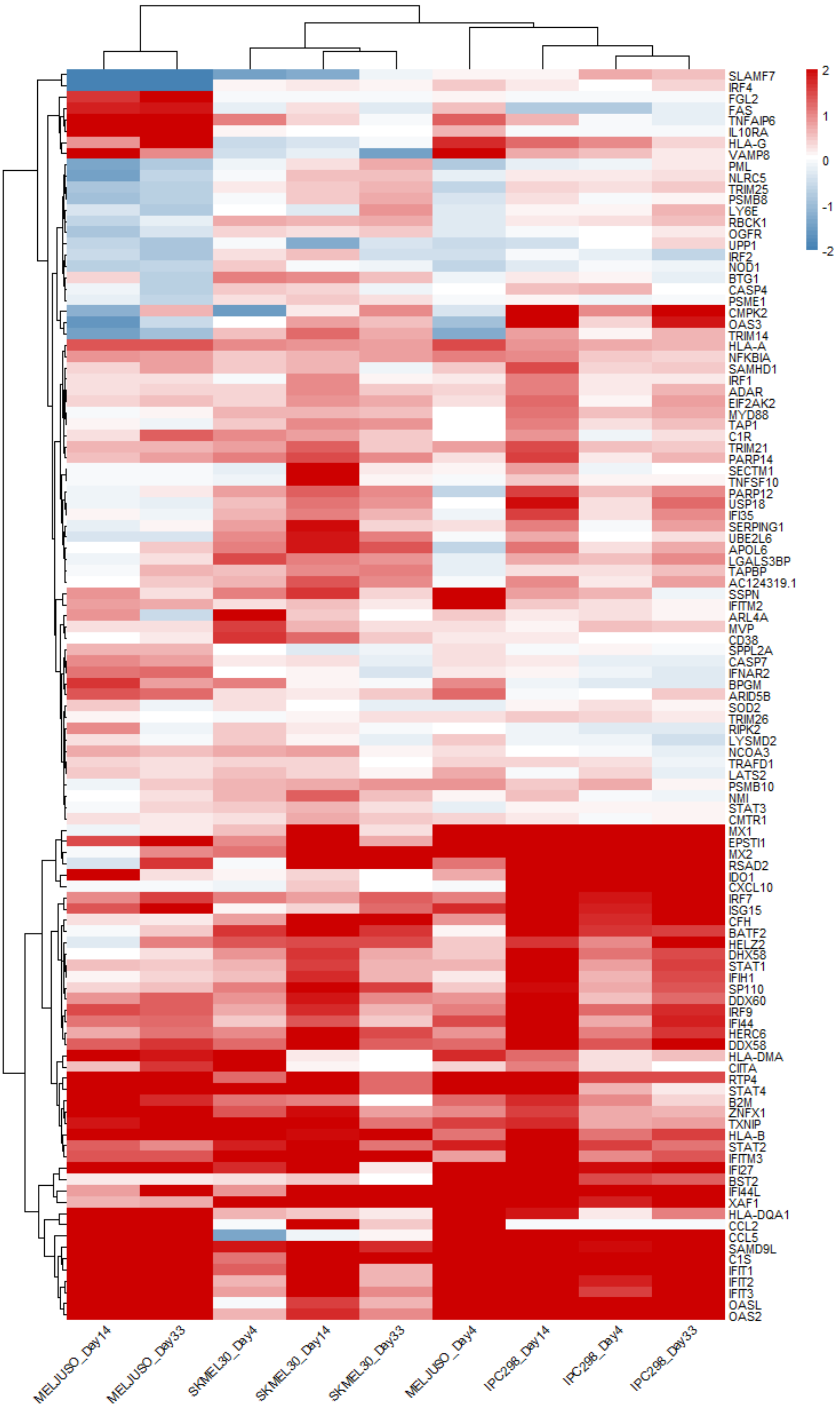

Supplementary Figure 22. Interferon Gamma response upon MEKi and CDK4/6i. The heatmap represents the logFC of the genes which contribute to the enrichment result. More precisely, it represents a sub-selection of the "INTERFERON GAMMA RESPONSE" gene set which contributes to the "leading edge" (ascendant portion) of the running sum used in GSEA.

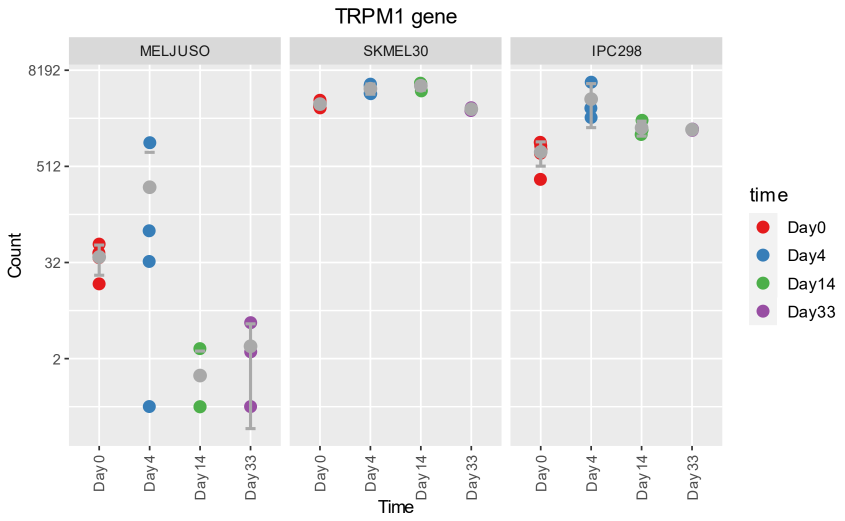

Supplementary Figure 23. Expression of TRPM1 transcripts in total RNA-seq. TRPM1 transcript contains miR-211-5p in an intronic region.

Supplementary Tables

	Gender	Stade	Mutations	Binimetinib (MEKi)	Palbociclib (CDK4/6i)	Phenotype
MELJUSO	Female	Primary	NRAS, TP53, CDKN2A, TERT, APC	110nM	1µM	Filliform
SKMEL30	Male	Metastatic	NRAS, HRAS	35nM	1µM	Pigmented
IPC298	Female	Primary	NRAS, TP53, CDKN2A, TERT	16nM	1µM	Normal
A375	Female	-	BRAF, CDKN2A, TERT	30nM	1µM	-
624MEL	Male	-	BRAF, TP53	50nM	1µM	-

Supplementary Table 1. Characteristics and concentrations of inhibitors used for the different cell lines across the study.

Gene	Total number of clusters enriched	Hierarchical clustering	k-medoids
NEAT1	8	3	5
GOLGB1	7	3	4
HMG20B	7	3	4
LUC7L3	7	3	4
MFSD12	7	3	4
S100B	7	3	4
TAX1BP1	7	3	4
TIMP2	7	3	4
ACTG1	6	3	3
ANXA5	6	3	3
B2M	6	3	3
BPTF	6	3	3
CANX	6	3	3
CD63	6	3	3
CHD4	6	3	3
CTSD	6	3	3
FTL	6	3	3
GNAS	6	3	3
GPNMB	6	3	3
HLA-A	6	3	3
HLA-C	6	3	3
HSP90B1	6	3	3
HSPA5	6	3	3
IGFBP7	6	3	3
KTN1	6	3	3
LGALS3BP	6	3	3
LMNA	6	3	3
NCL	6	3	3
PSAP	6	3	3
S100A6	6	3	3
SERF2	6	3	3
TMSB10	6	3	3
TOMM7	6	3	3
TUBA1A	6	3	3
UBC	6	3	3
YWHAB	6	3	3
ANXA2	5	3	2
CALR	5	3	2
CBX3	5	3	2
CCND1	5	3	2
CCT6A	5	3	2
DDX5	5	3	2
DSTN	5	3	2
EIF3A	5	3	2
FTH1	5	3	2
GAPDH	5	3	2
HLA-DRB1	5	3	2
HNRNPA2B1	5	3	2
HNRNPU	5	3	2
HSPB1	5	3	2
LIMA1	5	3	2
MAP4	5	3	2
MATR3	5	3	2
NDUFA4	5	3	2
PEBP1	5	3	2
PLP1	5	3	2
PRRC2C	5	3	2
RBM25	5	3	2
S100A10	5	3	2
SPTBN1	5	3	2
SQSTM1	5	3	2
SYNM	5	3	2
TPT1	5	3	2
YWHAZ	5	3	2
CAPN3	5	2	3
CDC37	5	2	3
HSP90AA1	5	2	3
HSP90AB1	5	2	3
AHNAK	4	2	2
AKAP12	4	2	2
CD74	4	2	2
CTNNA1	4	2	2
DAG1	4	2	2
HLA-B	4	2	2
HLA-DPB1	4	2	2
HLA-DRA	4	2	2
HSPG2	4	2	2
ITGA6	4	2	2
MCAM	4	2	2
MEGF9	4	2	2
MTDH	4	2	2
NQO1	4	2	2
PABPC1	4	2	2
PRNP	4	2	2
SNRNP35	4	2	2
TBC1D16	4	2	2
TMED9	4	2	2
WBP4	4	2	2
ZC3H13	4	2	2
ZNF121	4	2	2

Supplementary Table 2. 90 genes associated with senescence escape and numbers of clustersenriched for the two algorithms: hierarchical clustering and k-medoids.

Gene	Forward Primer (5' - 3')	Reverse Primer (5' - 3')	Size (bp)
NFIA	AGTGGATGGCATGAAGTGGA	CAGGGAGGAGGTCTGTGAAG	105
ILF2	CGTGGAAAGCCTAAGAGCAC	AAGATTGGGTGGCACTGTTG	127
TOP2A	CGCCCAGACACCTACATTG	TGTTTGTTGTCCGCAGCATT	155
LGALS3BP	TGAGTGTGGATGCTGAGTGT	CTTGACTGACGACAGGGTGA	94
CCND1	AGAGGCGGAGGAGAACAAAC	ATGGAGGGCGGATTGGAAAT	99
TXNIP	GAGCCAGCCAACTCAAGAGA	GAGCAGAGACAGACACCCGC	152
TOP1	GAATCAAGGGTGAGAAGG	GATGAAGTACAGGGCTAC	146
STAT2	GTTCTCCTCCTATGTTGG	CTCTCGCTTAGTGAAGTC	127
MYC	ACAGCATACATCCTGTCCGT	CGCACAAGAGTTCCGTAGC	119
MYO5A	CGGAAAGACCTGGAGCAAACTC	TGCTGCACGATGCGGTGATTGA	153
IFI6	GTGGCTGCCTCGCTGAT	TTACCTATGACGACGCTGCT	119
TYRP1	ACCAGAGGGTTCTCATAGTCAG	GCTTTGCCATATCCAGGG	90
**HPRT**	TGGACAGGACTGAACGTCTT	GAGCACACAGAGGGCTACAA	77
**PPIA**	CAGACAAGGTCCCAAAGACA	CCATTATGGCGTGTGAAGTC	139
**TBP**	ACCCAGCAGCATCACTGTT	CGCTGGAACTCGTCTCACTA	120

miRNA	Forward Sequence	ISB-UNI Sequence
miR-146a	UGAGAACUGAAUUCCAUGGGUU	ATCGAGCACCAGTTACGC
miR-211	UUCCCUUUGUCAUCCUUCGCCU	""
miR-320a	AAAAGCUGGGUUGAGAGGGCGA	""
miR-27a	UUCACAGUGGCUAAGUUCCGC	""
let-7a	UGAGGUAGUAGGUUGUAUAGUU	""
let-7f	UGAGGUAGUAGAUUGUAUAGUU	""

Supplementary Table 3. Primer list for mRNA and miRNAs measured by qPCR.
